# An integrated experimental–modeling approach to identify key processes for carbon mineralization in fractured mafic and ultramafic rocks

**DOI:** 10.1093/pnasnexus/pgae388

**Published:** 2024-09-18

**Authors:** Chelsea W Neil, Yun Yang, Haylea Nisbet, Uwaila C Iyare, Lawrence O Boampong, Wenfeng Li, Qinjun Kang, Jeffrey D Hyman, Hari S Viswanathan

**Affiliations:** Earth and Environmental Sciences Division, Los Alamos National Laboratory, Los Alamos, NM 87545, USA; Earth and Environmental Sciences Division, Los Alamos National Laboratory, Los Alamos, NM 87545, USA; Earth and Environmental Sciences Division, Los Alamos National Laboratory, Los Alamos, NM 87545, USA; Earth and Environmental Sciences Division, Los Alamos National Laboratory, Los Alamos, NM 87545, USA; Earth and Environmental Sciences Division, Los Alamos National Laboratory, Los Alamos, NM 87545, USA; Earth and Environmental Sciences Division, Los Alamos National Laboratory, Los Alamos, NM 87545, USA; Earth and Environmental Sciences Division, Los Alamos National Laboratory, Los Alamos, NM 87545, USA; Earth and Environmental Sciences Division, Los Alamos National Laboratory, Los Alamos, NM 87545, USA; Earth and Environmental Sciences Division, Los Alamos National Laboratory, Los Alamos, NM 87545, USA

**Keywords:** geologic carbon sequestration, carbon mineralization, subsurface flow, reactive transport modeling, geochemistry

## Abstract

Controlling atmospheric warming requires immediate reduction of carbon dioxide (CO_2_) emissions, as well as the active removal and sequestration of CO_2_ from current point sources. One promising proposed strategy to reduce atmospheric CO_2_ levels is geologic carbon sequestration (GCS), where CO_2_ is injected into the subsurface and reacts with the formation to precipitate carbonate minerals. Rapid mineralization has recently been reported for field tests in mafic and ultramafic rocks. However, unlike saline aquifers and depleted oil and gas reservoirs historically considered for GCS, these formations can have extremely low porosities and permeabilities, limiting storage volumes and reactive mineral surfaces to the preexisting fracture network. As a result, coupling between geochemical interactions and the fracture network evolution is a critical component of long-term, sustainable carbon storage. In this paper, we summarize recent advances in integrating experimental and modeling approaches to determine the first-order processes for carbon mineralization in a fractured mafic/ultramafic rock system. We observe the critical role of fracture aperture, flow, and surface characteristics in controlling the quantity, identity, and morphology of secondary precipitates and present where the influence of these factors can be reflected in newly developed thermo-hydro-mechanical–chemical models. Our findings provide a roadmap for future work on carbon mineralization, as we present the most important system components and key challenges that we are overcoming to enable GCS in mafic and ultramafic rocks.

**Significance Statement**

In the current study, we combine experimental and modeling approaches in geochemistry, geophysics, and subsurface flow to identify processes critical to the carbon mineralization in fractured mafic and ultramafic rock. This comprehensive analysis establishes a vital framework for understanding how carbon mineralization occurs in these systems, offering valuable insights for scientific understanding and practical applications in large-scale, long-term geologic carbon sequestration operations. These approaches are essential for enabling carbon sequestration in mafic and ultramafic formations, which can quickly react with carbon dioxide to form stable carbonate precipitates if challenges associated with their low porosity and permeability can be addressed.

## Introduction

To avoid the gravest consequences of climate change, the Intergovernmental Panel on Climate Change has recommended that atmospheric warming be kept below 2 °C (1). Meeting this goal not only requires immediate reduction of carbon dioxide (CO_2_) emissions, but also the active removal and sequestration of CO_2_ from current point sources (2). One of the most promising proposed strategies for CO_2_ storage is geologic carbon sequestration (GCS), whereby CO_2_ is trapped in the deep subsurface (3–5). However, there is currently a knowledge gap in our understanding of the geochemical reactions and mechanisms that stabilize stored CO_2_ through precipitation of CO_2_^−^ containing carbonate minerals. During GCS, CO_2_ is injected into subsurface formations, generally deep saline aquifers or depleted oil/gas reservoirs. To have a substantial impact on climate change mitigation efforts, GCS must reduce CO_2_ emissions on the scale of billions of metric tons (gigatons, Gt) per year (6). The US Department of Energy estimates that the CO_2_ storage capacity in these formations can be as high as 1,800 to 20,000 billion metric tons (7), equivalent to 600 to 6,700 years of current CO_2_ emissions from large stationary sources (8). GCS thus has the potential to revolutionize our approach to mitigating climate change. However, a lack of confidence in the long-term safety of stored CO_2_ remains a barrier to implementation (9).

After CO_2_ is injected into a subsurface formation, structural trapping by low-permeability caprock is typically the primary storage mechanism (10) until sufficient time has passed for CO_2_ to react in the subsurface, forming carbonate minerals (i.e. mineral trapping) (11). Mineral trapping is a much more stable storage mechanism as it does not rely on the structural integrity of the overlying caprock. To minimize risk, it is thus vital that subsurface CO_2_ be quickly mineralized into stable carbonates. In formations such as sandstone aquifers, which have abundant storage volumes and are widely available (3), mineralization is predicted to be insignificant until thousands of years after injection (12). By contrast, rapid mineralization in mafic and ultramafic rocks has recently been reported in both field and laboratory studies (13–18). Unlike sandstone and other felsic formations, mafic/ultramafic rocks, such as basalt and peridotite, have a significant pH buffer capacity, leading to the fast neutralization of acidic aqueous CO_2_ and facilitating carbonate precipitation (19). Mafic and ultramafic rocks are also abundant at the Earth's surface and near surface. Basaltic rocks make up about 10% of the continents and a significant portion of the ocean floor (13). These rocks are highly reactive in water and contain about 25 wt.% calcium, magnesium, and iron oxides (14), which combine with dissolved CO_2_ to form stable carbonate minerals.

Despite this high reactivity, mafic and ultramafic rocks (e.g. peridotite) have one key drawback: these formations are typically characterized by lower porosity and permeability (on the scale of ∼1–3% and 0.003–5 mD) (20, 21) relative to historically considered aquifer and oil/gas reservoir formations (on the scale of ∼20–30% porosity and ∼7–1,500 mD) (22). As a result, storage volumes and exposed reactive mineral surfaces are mostly limited to the preexisting fracture network. Storing the volumes of carbon necessary to combat global climate change thus necessitates sustainable reaction within fractured rock, meaning that it is critically important to prevent surface passivation and fracture clogging. Additionally, it is hypothesized that volume changes during mineralization can drive the formation of new fractures through reaction-driven fracturing (23). This mechanism, in addition to other means of expanding the fracture network, such as stress corrosion or subcritical fracturing (24), could be crucial in sustaining mineralization through increasing storage volumes and exposing new unreacted mafic/ultramafic rock surfaces.

Although CO_2_ mineralization has been studied for a long time (12, 25–29), understanding the feedbacks between geochemistry and geomechanics in fractures has recently become a topic of interest thanks to successful pilot-scale carbon mineralization operations at CarbFix (30, 31) and Wallula (32), which have proven that rapid mineralization is possible at some field sites (13). However, a fundamental understanding of the dominant processes in these complex coupled systems is needed to optimize and control mineralization at larger scales. These detailed mechanisms cannot be observed directly in the field, requiring in situ experiments under high pressure (P), temperature (T), and stress, as well as modeling, to understand their relative importance.

**Fig. 1.**
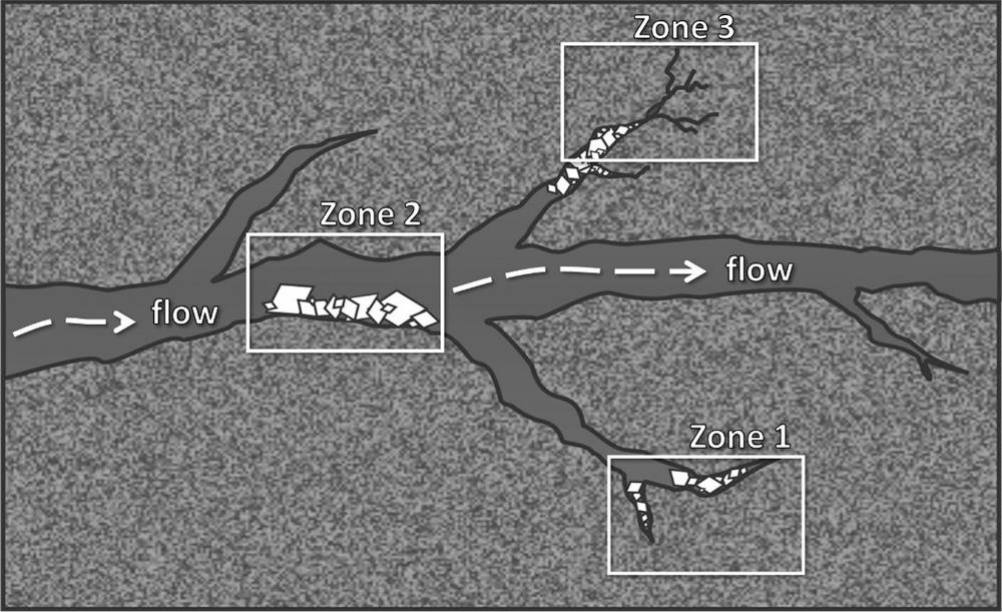
Schematic of carbon mineralization occurring in different zones of a fractured mafic/ultramafic rock.

Within a fractured, low-permeability rock, we have identified three distinct structural zones that are critical in determining the effectiveness of carbon mineralization as illustrated in Fig. 1. The first is dead-end fractures (Zone 1 in Fig. 1), areas of stagnant fluid flow where reaction is transport-limited. These zones have been shown to be sites of preferential carbon mineralization and may therefore provide the most potential for storage as carbonates (33, 34). The second zone is the primary flow pathway (Zone 2 in Fig. 1). These are regions where mineralization is a function of the reaction rate vs. the fluid advection rate. These are also zones where, if enough precipitation were to occur, a fracture could clog, reducing the overall storage volume and mineralization potential (35). The extent of clogging and its impact on the overall mineralization potential must be characterized for these geologic formations to understand what interventions may be needed for clogging prevention. The last zone is where fracture propagation could occur (Zone 3 in Fig. 1). These are dead-end fracture regions where the geometry of mineralizing carbonates is such that additional fractures may form, exposing fresh surfaces for mineralization and increasing potential storage volumes (36). Two important considerations for this mechanism are the kinetics of reaction and the spatial coupling of dissolution and precipitation. There is evidence from the literature on reaction-driven cracking during olivine hydration, indicating that dissolution and precipitation are collocated (37). If this is not the case for CO_2_ mineralization, it is unknown whether volume expansion can still drive cracking. It is also currently unknown whether cracking will occur on the timescale of GCS and whether cracking is necessary to achieve required storage volumes, considering the preexisting fracture network.

The goal of this paper is to utilize our unique integrated experimental and modeling approaches in the areas of geochemistry, geophysics, and subsurface flow, in addition to information from recent field studies (30–32, 38–40), to identify first-order processes that are most critical to the overall mineralization potential of fractured mafic and ultramafic rock within these three zones. For Zone 1, we undertake surface-sensitive geochemical characterization using Raman spectroscopy to observe how fracture characteristics (e.g. surface roughness and aperture) impact the extent of mineralization and formation of passivating layers. These dead-end fractures are ubiquitous in subsurface rock formations and have been shown to significantly impact solute transport processes (41, 42). For Zone 2, we employ a microfluidics system to observe coupled reaction and flow in a simple fracture network containing both dead ends and a primary flow pathway for natural rock samples. CarbFix (38, 39) and Wallula (32, 40) sequestration projects in porous basalt have more prevalent flowing pathways compared with less permeable mafic/ultramafic rocks, such as peridotite. While this system does not provide the minute controls on fracture characteristics achievable through our approach to Zone 1, it allows for the direct observation of transport and chemistry for realistic mineralization systems, comparison of reaction vs. transport (e.g. Damköhler number), and measurement of how reaction affects flow and can potentially lead to clogging. Lastly, for Zone 3, we seek to determine whether a self-sustaining fracture network can be created via reaction-driven fracturing. This process may be critical for sustainable mineralization in low-permeability rocks, such as those present in Oman ophiolite (43). To explore this mechanism, we are studying how the alteration of single olivine crystals can enhance fracture propagation through the formation of dissolution pits. The experimental apparatuses employed to study these zones work at the nexus of geophysics and geochemistry and allow for measurements at elevated T/P using real geomaterials. Experiments are complemented by lattice Boltzmann method (LBM) modeling, to determine reaction mechanisms, and discrete fracture networks (DFNs), to explore transport and reaction in scaled fracture networks. These modeling approaches allow us to extrapolate from smaller-scale studies to determine mineralization impacts at the field scale.

The observations and approaches presented in this paper begin to shed light on critical processes associated with carbon mineralization in fractured mafic and ultramafic rock including surface passivation and clogging, which can impact the sustainability of future storage operations including at larger scales. These approaches are critical to enable GCS in mafic and ultramafic formations, which have the potential for much faster mineralization if challenges associated with their low porosity/permeability can be overcome (43).

During carbon mineralization, divalent cations (Ca^2+^, Mg^2+^, and Fe^2+^), provided by the dissolution of silicate minerals, react with dissolved CO_2_ to produce carbonate minerals, such that CO_2_ is converted into a stable carbonate phase (44–46). To engineer this process, the relationship between the dissolution/precipitation rate and one or more independent variables (including temperature, ionic strength, the concentration of solute or surface species, and thermodynamic affinity) needs to be obtained. A general form of the rate law for dissolution–precipitation is given as follows (47):


(1) $$r=k_{0}A_{s}\exp\;\left(-\frac{E_{a}}{\mathrm{RT}}\;\right)a_{H^{+\;}}^{n_{H}}\prod_{i}a_{i}^{n_{i}}g(I)f(A),$$

where *r* is the reaction rate (mol × L^−1^×s^−1^), $k_{0}$ is the rate constant (mol × m^−2^×s^−1^), $A_{s}$ is the reactive surface area of the mineral exposed to the unit volume of aqueous solution (m^2^×L^−1^), $E_{a}$ is the activation energy of the dissolution/precipitation reaction (kJ × mol^−1^), $a_{H^{+}}^{n_{H}}$ characterizes the pH dependence of reaction rate, $\prod_{i}a_{i}^{n_{i}}$ accounts for possible catalytic or inhibitory effects associated with other solutes, and g(I) and f(A) measure the thermodynamic affinity that can be determined by Gibbs free energy.

The variation in the reaction rate with temperature is generally described by the Arrhenius equation, where the reaction rate constant increases exponentially with temperature (48). A transitional state theory (TST) -based rate law (49) can generally categorize the dependence of reaction rate on thermodynamic affinity and pH of the aqueous phase. The reaction rate is found to be linearly correlated with the square root of ionic strength in the log–log scale (49). As a result, attempts have been made to accelerate mineralization by enhancing the ionic concentration in the solution. For example, the CarbFix project adopted a novel injection system that predissolved CO_2_ into down-flowing water to artificially increase the ionic strength of carbonic acid, ultimately converting more than 90% of injected CO_2_ into calcite within 2 years (50). Alternatively, the reaction rate can be altered by increasing the reactive surface area and temperature. For this reason, preferred injection sites are deeper or within areas with a high geothermal gradient (51). Early field tests indicate that temperature trends observed in the laboratory may hold in the field. For example, CarbFix observed more than 90% mineralization in 2 years at ∼30 °C (50), while CarbFix2 observed more than 50% mineralization over 4 to 9 months at 250 °C (52). However, it is important to note that direct quantitative comparison of CarbFix and CarbFix2 mineralization rates is not possible due to the mineralization extents being measured at different times. Thus, these results may not capture changes in reaction rate that can occur with time due to mechanisms such as surface passivation.

Additionally, carbonate formation primarily occurs in pores and fractures with limited flow such as dead-end zones (33, 34), and thus, the reactant concentrations strongly depend on the distribution of pores and fractures and their flow conditions, in addition to dissolution and precipitation rates. Understanding the impact of the fracture and pore network on mineralization is therefore essential to understanding the mineralization process in mafic and ultramafic rocks. Nevertheless, limited bench-scale studies provide a quantitative determination of carbon mineralization rates within fractured mafic and ultramafic rocks, particularly those that specifically focus on a mechanistic understanding of how fracture characteristics (e.g. surface roughness, fracture aperture, fluid flow rates, and fracture connectivity) impact reaction. Such studies are critical to provide input and validate predictive pore/single fracture to fracture network-scale simulations of carbon mineralization in fractured mafic/ultramafic formations. The goal of our ongoing research effort is to provide these benchmark experimental datasets and link experiments with modeling.

## Zone 1: mineralization in dead-end fractures

Multiple studies of carbon mineralization by mafic/ultramafic rocks have shown that reaction preferentially occurs within reduced flow zones (i.e. Zone 1 in Fig. 1) such as dead-end fractures (34, 53, 54). Within these zones, mass transfer is controlled by diffusion rather than advection, i.e. the reactions are transport-limited as opposed to being kinetically limited (41, 55). As a result, fluid can become supersaturated with respect to precipitating carbonate minerals even while remaining undersaturated within flow channels (56). This process must be balanced with a continuous supply of reactants and is thus transport-limited in the case of carbonate mineralization, where CO_2_ is provided through injection. Compared with the primary flow pathway, where dissolution dominates over precipitation, sustained reactions can occur within dead-end fractures. This is because when dissolution dominates, silica-rich passivating layers form, likely due to the preferential release of divalent cations, halting further reaction (57). Thus, it is critically important to understand the mineralization potential within these zones to assess overall carbon storage in mafic formations.

One area where knowledge is lacking is the impact of fracture characteristics, such as surface roughness and fracture aperture, on precipitation within these zones. While surface roughness effects on carbon mineralization for basalt have not been reported on in the literature, previous studies of calcite epitaxial nucleation and growth on smooth vs. abraded calcium carbonate single crystals showed that rougher surfaces promote more nucleation while smooth surfaces promote a single crystal overgrowth layer (58). Similarly, the effects of fracture width on carbon mineralization have not been systematically explored for mafic/ultramafic rocks. It has been reported that larger fracture apertures promote dissolution over precipitation, as diffusive transport of reactants, such as CO_3_^2−^, to the mineral surface is slower (59).

**Fig. 2.**
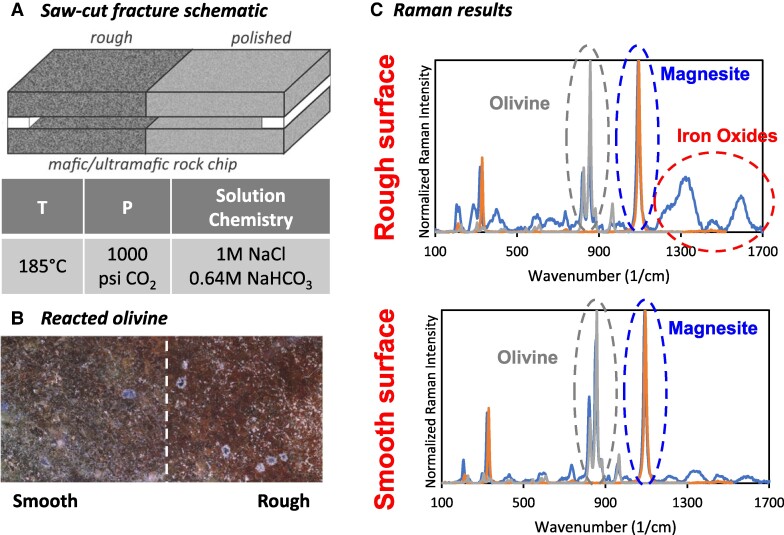
A) Experiment schematic and reaction conditions; B) optical microscopy image of reacted olivine; and C) Raman spectra for the smooth and rough internal surface of a reacted olivine fracture. Peaks due to increased iron oxide precipitation on the rough surface occur between 1,200 and 1,700 wavenumbers. These iron oxides reduce available divalent cation concentrations and could passivate the reactive surface.

We conducted preliminary experiments using saw-cut olivine ((Mg, Fe)₂SiO₄) fractures with controlled surface roughness and fracture width to begin probing these outstanding questions. Artificial fractures, created by sandwiching two rock wafers together, were reacted using a Series 4560, 300 mL Parr vessel (Parr Instrument Company, Moline, IL, USA) at conditions chosen to promote mineralization (Fig. 2A). Post-reaction characterization was conducted using a Horiba Jobin Yvon Evolution Raman spectrometer (Horiba, Ltd, Kyoto, Japan).

Figure 2B presents the optical microscopy image, and Fig. 2C presents Raman spectra for the rough and smooth surface inside of a 0.6-mm-wide fracture. There is a clearly visible line (indicated by dashed line in Fig. 2B) in the optical microscopy images showing more reddish-colored precipitation on the rough surface. This was confirmed using Raman spectroscopy, which showed that more iron oxides (maghemite, γ-Fe_2_O_3_) precipitated on the rough surface, as indicated by the broad Raman peaks between 1,200 and 1,700 cm^−1^ (60). Although the system was purged with CO_2_ to minimize O_2_ in the water, hydrolysis of water has been reported to trigger iron oxide formation during olivine carbonation (61). Both surfaces also had detectable carbon mineralization in the form of magnesite (MgCO_3_). These results show that surface roughness is an important parameter when considering the carbon mineralization mechanism within fractures. In particular, the formation of iron oxides on rougher surfaces can negatively impact long-term storage potential by both passivating reactive mineral surfaces and reducing the concentration of reactive cations (e.g. Fe), which could alternatively precipitate iron carbonates.

We are complementing these results with single fracture models looking at passivation and surface roughness effects. We performed pore-scale LBM simulations (62) of diffusion–reaction processes involving dissolution and precipitation using a simple but general chemical model:


(2) $$A_{\left(\mathrm{aq}\right)}+D_{\left(s\right)}⇆B_{\left(\mathrm{aq}\right)}\mathrm{and}$$

(3) $$B_{\left(\mathrm{aq}\right)}+C_{\left(\mathrm{aq}\right)}⇆P_{(s),}$$

where (aq) stands for the aqueous species and (s) denotes the solid phase. Eq. (2) is the dissolution reaction in which aqueous A_(aq)_ reacts with solid D_(s)_, generating aqueous B_(aq)_. The generated B_(aq)_ reacts with another aqueous C_(aq)_, producing the secondary precipitate P_(s)_ according to precipitation reaction Eq. (3).

**Fig. 3.**
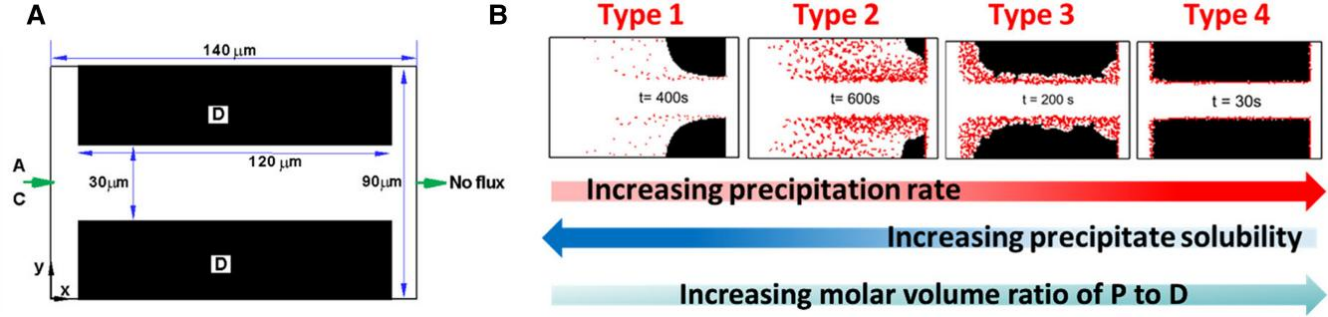
A) Schematic of the computational domain. Concentration is fixed at the left inlet and concentration gradient is set to zero at the right outlet with a zero injection velocity. B) Four types of coupled dissolution–precipitation processes. Adapted from Chen et al. (62) and reprinted with permisson from Elsevier.

The 2D computational domain is a rectangular block of solid D_(s)_ with a straight channel in the middle, as shown in Fig. 3A. The initial solution in the channel contains only A_(aq)_ and B_(aq)_ in equilibrium with D_(s)_. Then, a solution containing only A_(aq)_ and C_(aq)_ is introduced at the left inlet. The introduction of this solution, with concentration C_A, in_, impacts the initial equilibrium among A_(aq)_, B_(aq)_, and D_(s)_. Because C_A, in_ is higher than the initial concentration of A_(aq)_, D_(s)_ starts to dissolve at the D_(s)_–fluid interface according to Eq. (1). B_(aq)_ is thus generated, and its concentration increases. It further reacts with C_(aq)_ from the inlet and produces the precipitate P_(s)_. The precipitation occurs at a surface node or a stable nucleus once the solution there becomes supersaturated with respect to the precipitating phase. Thus, the precipitation and dissolution reactions are coupled with each other. In addition, the generated precipitates may cover the surface of D_(s)_ and affect subsequent mass transport and dissolution processes. The simulation includes a barrier to the first precipitation defined as the crystal growth threshold. For our simulations, the energy barrier was kept constant for the initial solid surface, D(s), and precipitating phase, P_(s)_, to limit the variables being explored. Transport in the model is driven by the concentration differences between the left inlet and right outlet (Fig. 3A), meaning that movement is driven by diffusion rather than advection. Since fluid flow is not considered in the simulation, the simulation system mimics a dead-end fracture with a geometry similar to our experimental work.

Using this model, we systematically investigated the effects of precipitation on dissolution under different dissolution–precipitation reaction kinetics, molar volumes of both solid phases, grain size, surface roughness, and nucleation and crystal growth mechanisms. Different morphologies of the precipitates are predicted by these pore-scale simulations. It is found that precipitation has opposing effects on the underlying dissolution. The favorable effect is that the precipitation reaction consumes the product of the dissolution reaction, thus facilitating further dissolution, while the unfavorable effect is that the generated precipitates cover the surface of the primary solid phase, thus isolating the reactive surface from the reactive components. Based on the extent to which the precipitates affect dissolution, we identified four types of coupled dissolution–precipitation processes as shown in Fig. 3B (62). The reactions considered in these systems are generic, and parameter values were not chosen to match any physical systems. Rather, this simulation is a parametric study investigating the relative effects of different key parameters (e.g. reaction rate constant, equilibrium constant/solubility, and molar volume).

In Type 1, the amount of generated precipitate is minimal due to low precipitation reaction rate, high solubility, or small molar volume of the precipitate, such that the primary solid phase D_(s)_ is completely dissolved. However, extensive precipitation is not achieved. In Type 2, more precipitates are generated; but they are too porous to inhibit the transport of reactant such that D_(s)_ can still be completely dissolved. For this type, the favorable effect of precipitation on dissolution dominates and dissolution is accelerated. This type leads to both maximum precipitation and dissolution and is the most favorable type for carbon mineralization. Further increasing the precipitate reaction rate, decreasing the solubility of the precipitate, or raising the molar volume of the precipitate leads to Type 3. In this type, precipitates are sufficiently compact that they can stop the dissolution completely (i.e. passivate the surface) and dissolution is only allowed to a somewhat limited extent. In Type 4, precipitation is very fast (30 s vs. 200–600 s for Types 1–3) and the precipitates are extremely compact, coating the entire surface of the primary solid phase and completely stopping the dissolution more quickly than in Type 3. In this type, the unfavorable effect of precipitation on dissolution overwhelms the favorable effect. In both Type 3 and Type 4, the entire surface of the primary mineral is eventually coated.

In line with our experimental results, we used this simplified model to study the effect of the initial surface roughness of the solid phase, D_(s)_, on the relative balance of dissolution and precipitation for the four types of systems. Surface roughness is generated by arranging rectangular protuberances on the grain surface. The general evolutions of geometries of D_(s)_ and P_(s)_ are similar to those without surface roughness, and the four types of dissolution–precipitation processes are generally maintained for Types 1, 2, and 4. However, roughness has a significant effect on the extent of dissolution and precipitation for Type 3, in which both the rate and amount of dissolution and precipitation increase with the initial surface roughness due to increased reactive surface area. This is consistent with our experimental observations of more extensive iron oxide precipitation on rougher surfaces, indicating that olivine dissolution and secondary mineral precipitation may fall into this Type 3 category. However, this simplified model does not capture differences in heterogeneous precipitation behavior between carbonate and iron oxide precipitates. In our experiments, the relative quantities of carbonate precipitates did not appear to have the same dependence on surface roughness as iron oxides. Thus, reaction-specific complexities must be captured through more detailed models aimed at matching the system chemistry.

**Fig. 4.**
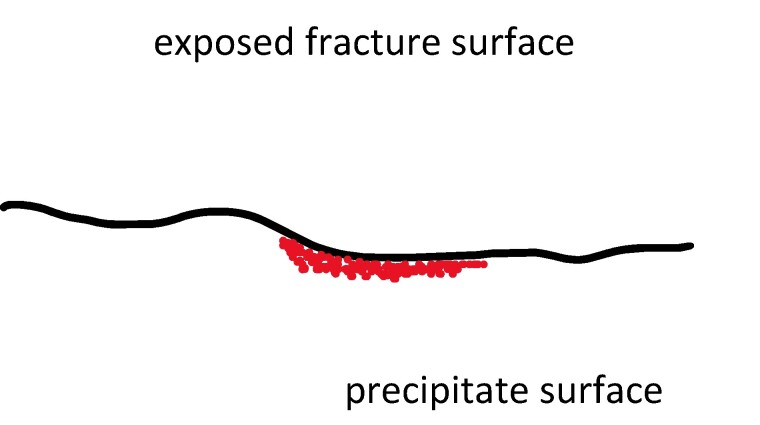
Schematic of passivation effects on reactive surface area of the dissolving mineral. The exposed surface area of the fracture corresponds to the total surface area including the region that is in contact with the precipitate.

Our detailed experiments and pore-scale modeling indicate that passivation is a first-order process that should be considered in reservoir-scale models. We are working toward this goal using a reaction model that has been recently implemented in the PFLOTRAN simulator (63). This model calculates the effective surface area of a dissolving mineral available for reaction due to the passivating effect. The model assumes heterogeneous nucleation for the precipitating mineral and calculates the total exposed surface area of the dissolving mineral and the surface area of the precipitating mineral that is in contact with the dissolving mineral (Fig. 4). These parameters are used to calculate the effective surface area of the dissolving mineral and can account for the different dissolution and precipitation rates of different mineral phases. The conductivity of the precipitate (i.e. either porous or nonporous) is also considered in the model and can thus account for the effects of the Types 1–4 systems as outlined above.

We validated the passivation model within PFLOTRAN using experimental wollastonite carbonation data (64). The model predicted that calcite passivation on the wollastonite surface minimizes the carbonation extent, which is consistent with experimental outcomes. The passivation model is being incorporated into single fracture and DFN models. As informed by both our recent experimental explorations and the results from corresponding LBM simulations, the connection between passivation and surface roughness needs to be a focus of study using these new models. Recent advances in 3D DFN simulations allow surface roughness to be included in reactive transport simulations (65–67). A highly refined computational mesh can also be used to include aperture variables sampled from experiments at relevant length scales (41). Furthermore, the roughness can dynamically evolve in space and time within these modern DFN models, just as they will in the real system (41). This ability to include the effect of fluid–mineral reactions on spatially variable hydraulic resistance will provide multiple avenues to address long-standing questions related to the role of surface passivation in inhibiting long-term mineralization within fractured media.

## Zone 2: mineralization in primary fracture pathways

Fractures in low-permeability rocks, e.g. mafic and ultramafic rocks, are the preferential pathways for fluid flow and mass transport in the subsurface (68). Within these primary flow zones (i.e. Zone 2 in Fig. 1), the rate of mineralization is primarily controlled by the rate of the geochemical reaction and the advection rate, with diffusion expected to play a smaller role (69). Many experiments aimed at understanding carbon mineralization in mafic and ultramafic rocks are conducted in static systems, where hydrodynamic processes are not considered (70–73). Moreover, efforts to understand fluid dynamic processes in flowing fractures frequently exclude chemical reactions or solely investigate one reaction (dissolution or precipitation, rather than both). Given that CO_2_ mineralization in mafic and ultramafic rocks involves both chemical and mechanical processes, the development of experiments that couple dissolution and precipitation is advantageous to bridge this knowledge gap and develop a further understanding of the storage capacity of a given system.

A key uncertainty that remains in our understanding of mineralization in a flowing fracture environment is the interplay between dissolution and precipitation. It is important to know how these reactions are coupled because they will ultimately define the CO_2_ storage capacity of a reservoir and play a fundamental role in the mechanical properties. For example, the location where dissolution and precipitation occur in a fracture network can control whether a pathway will clog, reducing the accessible volume for fluid transport and mineralization, or whether the precipitation of minerals could result in the propagation of fractures, increasing storage capacity.

Previous experimental studies suggest that mineralization is favored in diffusion-dominated regions and advection-dominated regions are defined by dissolution. Flow-through microfluidics experiments conducted by Menefee et al. (34) in basalt demonstrated preferential dissolution in primary fluid pathways, whereas mineralization occurred in the dead-end zones. This was similarly observed by Andreani et al. (57) in sintered dunite where, in addition to dissolution, the authors also observed the formation of a passivating Si-rich layer. However, natural examples of carbon mineralization, such as in Oman's ophiolite, depict widespread mineralization, including within primary fractures (74).

**Fig. 5.**
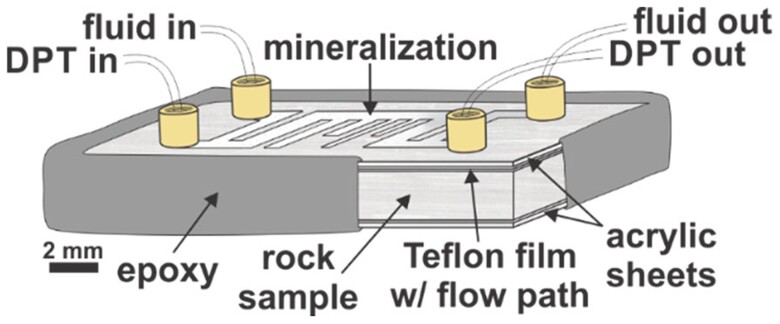
Schematic of microfluidics model used to investigate carbon mineralization in a flowing fracture.

Microfluidics experiments were carried out using a natural rock analog system to investigate the extent and spatial distribution of dissolution and precipitation during carbon mineralization at various advection rates (flow rates). The reaction of gypsum to calcite was selected due to the relative abundance of available literature and its fast reaction rate. Rectangular chips of crystalline gypsum (selenite, Ward’s Scientific) were cut, and a comb-like flow path was defined by a laser-cut Teflon sheet, sandwiched between acrylic sheets, and held together by epoxy (Loctite metal/concrete) (Fig. 5). The assembled micromodel was placed inside of a high P/T optical cell and flushed with a carbonated solution at various flow rates under 1,500 KPa confining pressure at ambient temperature for 4.5–5.5 h. Surface profilometry and scanning electron microscopy (SEM) analyses were carried out to characterize the changes in surface morphology and mineralogy.

**Fig. 6.**
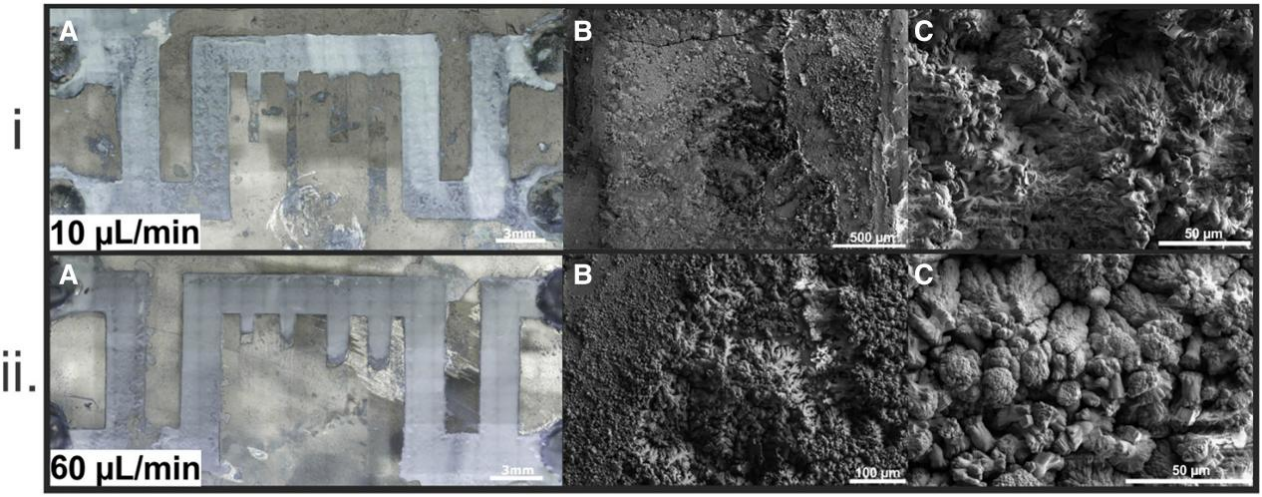
Post-experiment images of microfluidics experiments run at 10 µL/min (i) and 60 µL/min (ii). A) Profilometry scans of selenite chips post-experiment showing the spatial distribution and pattern of precipitation within the channel. B) and C) are images obtained from SEM analyses showing the various textures of the precipitated minerals vaterite and calcite. The faster flow rate increased the proportion of calcite to vaterite, which may impact clogging due to the differences between polymorph structure and density.

Experimental results depict a visible difference in the mineralization within the main channel at different flow rates. As shown in Fig. 6i, at a flow rate of 10 μL/min, precipitation occurred uniformly throughout the main channel, resulting in a porous texture. As the flow rate increased to 60 μL/min, precipitation predominated further down in the channel, while dissolution was focused at the inlet. The resultant carbonate precipitation was also less porous (Fig. 6ii). SEM analyses identified the reaction product as primarily the metastable calcium carbonate mineral, vaterite, with the more thermodynamically stable calcite disseminated throughout the sample (< 10%). Notably, at faster flow rates, calcite was more dominant. None of the experiments exhibited channel clogging, suspected to be caused by the reaction of gypsum to calcite and vaterite being a volume reduction reaction. The spatial decoupling of dissolution and precipitation with higher advection rate may be due to the higher gypsum dissolution rate in higher advective flows, where the reaction is surface-controlled (75). This results in an excess of Ca^2+^ ions released and transported further down the channel, eventually precipitating as calcium carbonates. Thus, higher flows lead to the precipitation reaction front moving further from the injection point. This can also lead to higher degrees of supersaturation, kinetically favoring vaterite and promoting a faster transformation to calcite (76).

A noticeable difference was also observed in the amount and type of precipitation found in the dead-end channels of the flow path, where the faster flow rate resulted in more mineralization within the dead ends and a greater abundance of calcite. Current experiments cannot reveal whether the calcite forms as a result of vaterite transformation or direct precipitation. This mechanism will be explored through future time series experiments. We also plan to conduct similar experiments to investigate the reaction of calcite to gypsum (the reverse reaction), which is governed by an increase in volume, to investigate the potential for channel clogging as a function of advection rate. Furthermore, we will shift toward more realistic systems by reacting mafic and ultramafic rocks collected from pilot-scale operations (e.g. CarbFix, Oman) at optimal carbonation conditions. Coupled with numerical modeling studies, we aim to delineate the primary factors contributing to CO_2_ mineralization in a flowing fracture toward the goal of determining optimal injection conditions for CO_2_ mineralization operations.

**Fig. 7.**
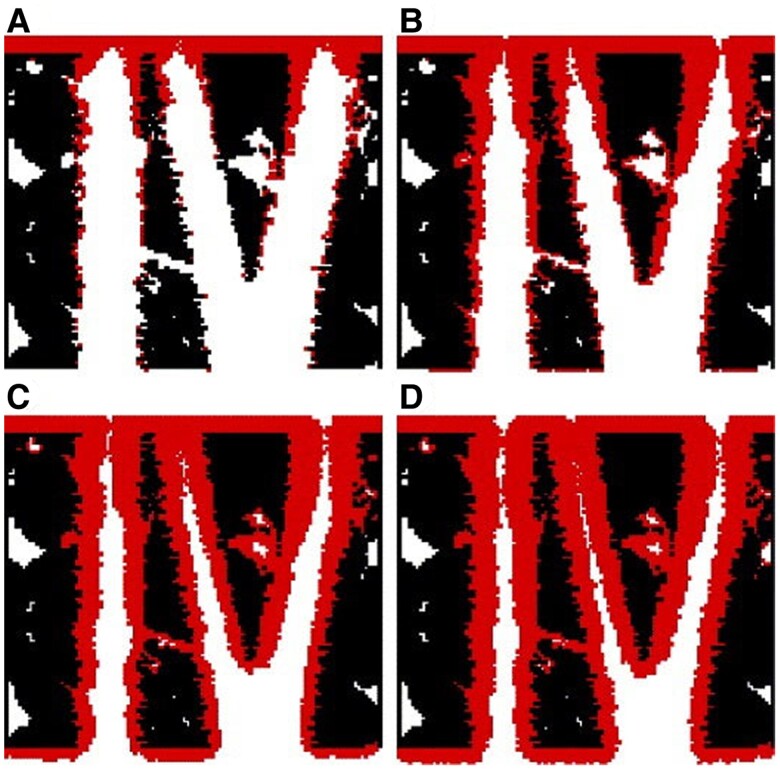
Simulated coupled flow, solute transport, and precipitation at a fixed Péclet number (Pe) but decreasing Damköhler number (Da) from A) to D). From Kang et al. (77) and reprinted with permission from Elsevier.

LBM pore-scale simulations of coupled fluid flow, solute transport, and precipitation give insight into the phenomena observed in microfluidics experiments (77). In the simulation, a general first-order kinetic reaction model at the fluid–solid interface is considered. Initially, the pore space is filled with a solution in equilibrium with the solid (black in Fig. 7). Then, an oversaturated solution is introduced at the entrance (top), and the precipitation (red) occurs along the fracture wall. As shown in Fig. 7, at a high Damköhler number, precipitation mainly occurs at the entrance, resulting in quick clogging of the fractures and halting fluid flow, mass transfer, and further precipitation. As Damköhler decreases, more precipitation takes place downstream. A similar trend is found with a fixed Damköhler number and an increasing Péclet number. These scenarios represent slower flow (Fig. 7A) vs. faster flow (Fig. 7D) and align with observations of more precipitation downstream for the 60 μL/min microfluidics experiment. These results highlight the crucial interplay between transport and reaction. However, current models do not capture the different potential polymorphs of calcium carbonate that can form, as observed in experiments. Incorporating these polymorphs requires knowledge of the formation kinetics, properties, and stability of these phases, some of which require experimental determination. The morphological differences between these polymorphs and their different surface densities may be an important consideration when predicting whether a flowing channel will clog and whether a reactive surface will become passivated. These experimental findings on simultaneous precipitation/dissolution systems are also being put into PFLOTRAN to study their impact on mineralization at the reservoir scale. Further experiments will inform whether the observed differences in calcium carbonate polymorphs should also be included in these models.

## Zone 3: fracture propagation due to reaction-driven cracking

It is currently unanswered whether mineralization is scalable if we rely only on the existing fracture network. In addition to exploring this question, we must investigate whether it is possible to increase the fracture network through reaction-driven cracking or stress corrosion (subcritical cracking). Carbon mineralization in mafic and ultramafic rocks can be self-limiting or self-sustaining depending on the relative strengths of competing positive and negative feedbacks, driven by the mineralization reaction. Two processes are responsible for negative feedbacks, as documented in prior dead-end and flow-through reactive transport experiments. First, precipitates of product minerals can clog hydraulic pathways and reduce porosity and permeability, limiting fluid supply to reaction zones (78, 79). Second, mineral precipitation can coat the surface of CO_2_^−^reactive minerals, inhibiting further reaction (55). On the other hand, two positive feedbacks can sustain carbon mineralization until completion. One is reaction-driven cracking (Zone 3 in Fig. 1), proposed by Macdonald and Fyfe (80), who hypothesized that stress induced by volume expansion and mineral transformation during carbonation is large enough to grow cracks, thus increasing permeability and exposing new reactive surfaces (67, 81–83). The other is that mineral dissolution increases exposed unreacted surface areas via etch pitting and channel formation to promote further reaction (84, 85).

Petrographic observations support the hypothesis of reaction-driven cracking. Dense fracture systems, filled with secondary product minerals, have been observed in outcrops (83, 86), suggesting a coeval process of cracking and carbonation. However, there is no consensus on the mechanism by which hydration- or carbonation-induced volume expansion increases permeability and reactive surface area. Crystallization pressure has been hypothesized as the cause of reaction-driven cracking (75). Others hypothesize that the nonuniform volumetric strain (eigenstrain) caused by mineral transformation is responsible for reaction-driven cracking (87). Multiphysical modeling, despite differences in assumptions and approaches, demonstrates that the extent of reaction is critically controlled by crack growth resulting from nonuniform solid mass and volume increases after reaction (81, 83, 88, 89). Despite these proposed mechanisms, there are sparse publications directly observing reaction-driven cracking or reporting conditions for cracking optimization (37, 87).

We hypothesize that an initial hierarchical crack system, in addition to temperature, pressure, fluid chemistry, flow rate, geochemical reactions, etc., can promote reaction-driven cracking in mafic and ultramafic rocks during carbonation. The hierarchical system refers to a combination of rough-walled main fractures, where fast fluid flow and advective transport dominate, and secondary fractures, where slow fluid flow and concentration-driven diffusion dominate, similar to the geometry explored through the microfluidics setup. Fluid flow in the main fracture may also supply mineral precipitates that can be deposited at the mouth of secondary fractures, especially considering the roughness of fracture walls. Mineral precipitation can further grow in the secondary fractures, either encouraged by local fluid chemistry or nonequilibrium mineral phases, propagating the expansion of secondary fractures through reaction-driven cracking. Both crystallization pressure and nonuniform volumetric strain during reaction likely contribute to crack growth. Our previous work has demonstrated that mineral precipitation strongly correlates with the structural and geometrical details of fractures (90).

**Fig. 8.**
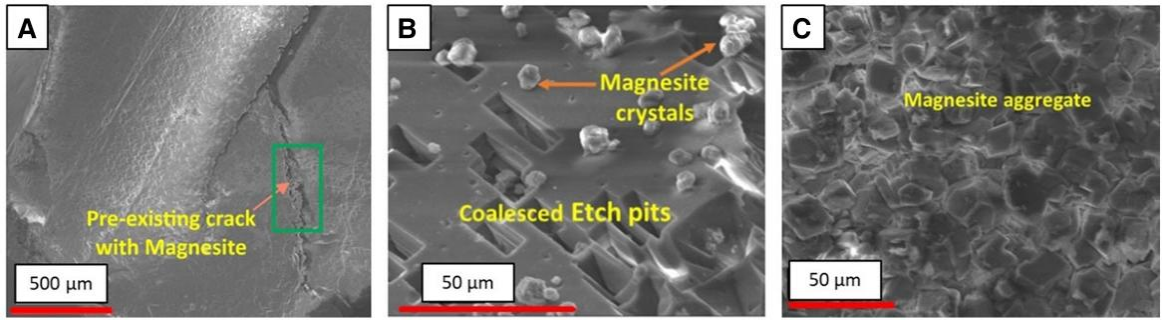
MgCO_3_ precipitating on reacted olivine shows A) formation within fractures, B) etch pit formation in systems further from equilibrium, and C) denser precipitates for systems close to equilibrium. These etch pits will increase reactive surface area and may act as a weak point for reaction-driven cracking.

To begin exploring this complex system, we conducted batch experiments looking at the morphological evolution of single grains of olivine with preexisting cracks. We made some important observations to support our hypothesis, such as the precipitation or gathering of MgCO_3_ precipitates within the fracture opening (Fig. 8A). Interestingly, we also observed the formation of extensive dissolution pits within systems further from thermodynamic equilibrium (Fig. 8B), while for systems closer to equilibrium, precipitation appeared to dominate the surface morphology (Fig. 8C). We hypothesize that the dissolution behavior further from equilibrium may promote fracture formation by providing resistance against precipitates growing within these pits. If the pressure exerted by these growing precipitates exceeds a critical force, the rock may fracture. For the etch pit geometry, this force can be calculated using the relationship (91):


(4) $$F_{c}=\frac{K_{\mathrm{Ic}}\sqrt{D}}{1.46},$$

where *F_c_* is the force per unit width perpendicular to the plane, *K*_lc_ is the intensity factor, and *D* is the pit depth. The force is then related to the pressure for fracturing according to the relationship:


(5) $$P_{c}=\frac{F_{c}}{L_{m\;}},$$

where *L*_m_ is the characteristic size of the precipitating minerals. Using the dimensions observed for our experiments, *P_c_* was estimated to be on order of 100 MPa, below the olivine carbonation crystallization pressure, reported by Kelemen and Hirth (82) to be up to 1.3 GPa based on our experimental conditions. Thus, fractures could be propagated from these etch pits, supporting the sustainability of storage operations. The propagation of these fractures is intricately linked to the hydrodynamics of the system, as they require precipitates to form at the tip of the existing fracture or etch pit. Experiments and modeling of Zones 1 and 2, as outlined in this study, can begin to inform to what extent precipitates will form in these locations based on solution chemistry, surface properties, and flow rates. We must explore how this process can be incorporated into THMC models to accurately capture the evolution of storage volumes and reactive surface area.

## Modeling geochemical reactions within fracture networks

To make defensible predictions of carbon mineralization at the field scale, laboratory observations of dead-end, flowing, and reaction-driven cracking effects and the associated mechanisms identified and quantified through pore- and fracture-scale modeling must be propagated into network-scale (hectometer) models. Previously, simulations of geochemical reactive transport through fractured media have mostly been performed in a single fracture, 2D networks, or in upscaled/equivalent continuum models (92–103). However, the interplay of length scales in 3D systems leads to multiscale flow heterogeneity within a fracture system. Such variation in the flow field cannot be adequately represented by 2D or continuum models. Thus, high-fidelity 3D simulations are sought after, but they are limited in scope and application due to computational expense. Recent developments in high-performance computing are allowing research teams to explore the interplay of geo-structural attributes, topology, geometry, and hydrological attributes, with flow and reactive transport properties in 3D fractured media (41).

In this section, we demonstrate new dynamic simulations of gypsum dissolution leading to calcite precipitation in 3D DFNs, matching the simpler surrogate system used in microfluidic experiments. Our overarching goal is to study the interaction of fracture network properties with coupled dissolution and precipitation leading to the accumulation of mineralized carbon. However, it is difficult to find a chemical system that has sufficiently rapid kinetics for both dissolution and precipitation, such that experiments can be conducted at laboratory time frames to benchmark our simulations. The kinetics also needs to be sufficiently rapid that enough dissolution and precipitation happen to significantly impact the hydraulic resistance offered by fractures. The dissolution and precipitation of both calcite and gypsum fit this criterion. Thus, we use gypsum dissolution leading to calcite precipitation as an analogous system for carbon mineralization to characterize the interplay of fracture network structure, flow dynamics, and geochemistry on mineral precipitation.

The geochemical formulation is the following. Gypsum dissolves when the mineral contacts a solution which is undersaturated. The gypsum dissolution reaction is governed by:


$$\mathrm{CaS}O_{4}.2H_{2}O(s)↔Ca^{2\;+\;}+{\mathrm{SO}}_{4}^{2-}+2H_{2}O.$$

Similarly, the calcite precipitation reaction is described by:


$$Ca^{2\;+\;}+{\mathrm{CO}}_{3}^{2\text{-}}↔\;\mathrm{CaC}O_{3}(s).$$

The mineral reaction rates for gypsum dissolution and calcite precipitation are calculated using the transition state theory (104):


(6) $$r_{m}=-A_{m}k_{m}(1-K_{m}Q_{m});$$

(7) $$Q_{\mathrm{gypsum}}=\;a_{Ca^{2\;+\;}}\times \;a_{{\mathrm{SO}}_{4}^{2\text{-}\;}\;}\times a_{H_{2}O}^{2};\;\mathrm{and}$$

(8) $$Q_{\mathrm{calcite}}=\;a_{Ca^{2\;+\;}}\times \;a_{{\mathrm{CO}}_{3}^{2\text{-}}\;},$$

where $r_{m}$ is the reaction rate for mineral m *(*mol/s m^3^), $k_{m}$ denotes the reaction rate constant *(*mol/s m^2^), $K_{m}$ denotes the equilibrium constant, and $Q_{m}$ is the ion activity product of mineral m (assumed unit water activity). $A_{m}$ represents the specific surface area of the mineral (m^2^/m^3^). It is important to note that *K*_m_ is for the precipitation reaction.

The reaction is classified as a precipitation reaction when the reaction rate is positive, and this occurs when $K_{m}Q_{m}>1$. The reaction, however, is in equilibrium when $K_{m}Q_{m}=1$, and the mineral dissolves when $K_{m}Q_{m}<1$, producing a negative rate.

The simulated chemical system comprises a fractured rock that initially contains only gypsum. This rock is saturated with a solution that is in equilibrium with the gypsum mineral. Na_2_CO_3_ solution was then injected into the rock. The injected solution is undersaturated with gypsum, causing the gypsum to dissolve (Table 1). The released $Ca^{2\;+\;}$ ion reacts with the ${\mathrm{CO}}_{3}^{2\text{-}}$ ion contained in the injected brine to produce calcite.

**Table 1.** Solution chemistry for fracture network modeling.

Ion	Initial brine	Injected brine
$Ca^{2\;+\;}$	$15.648\times 10^{-3}$ M	0.0
${\mathrm{SO}}_{4}^{2\text{-}}$	$15.648\times 10^{-3}$ M	0.0
${\mathrm{CO}}_{3}^{2\text{-}}$	0.0	0.5 M
$Na^{+\;}$	0.0	1.0 M
pH	7.07	11.39

For our simulations, we consider pressure-driven flow through a 3D DFN. The DFN is generated using the dfnWorks software suite (105), which creates a high-resolution computational mesh of the fracture network (106, 107). This mesh is critical because it allows for spatially variable dissolution and precipitation of minerals within each fracture in the network. The 3D DFN is generic, being composed of monodisperse square fractures with edge ledges of 2 m and uniformly random orientations, thereby representing a strongly disordered media (108). Reactive flow and transport simulations are performed using the massively parallel reactive flow and transport simulator PFLOTRAN (63). The simulation was run for 10 days.

**Fig. 9.**
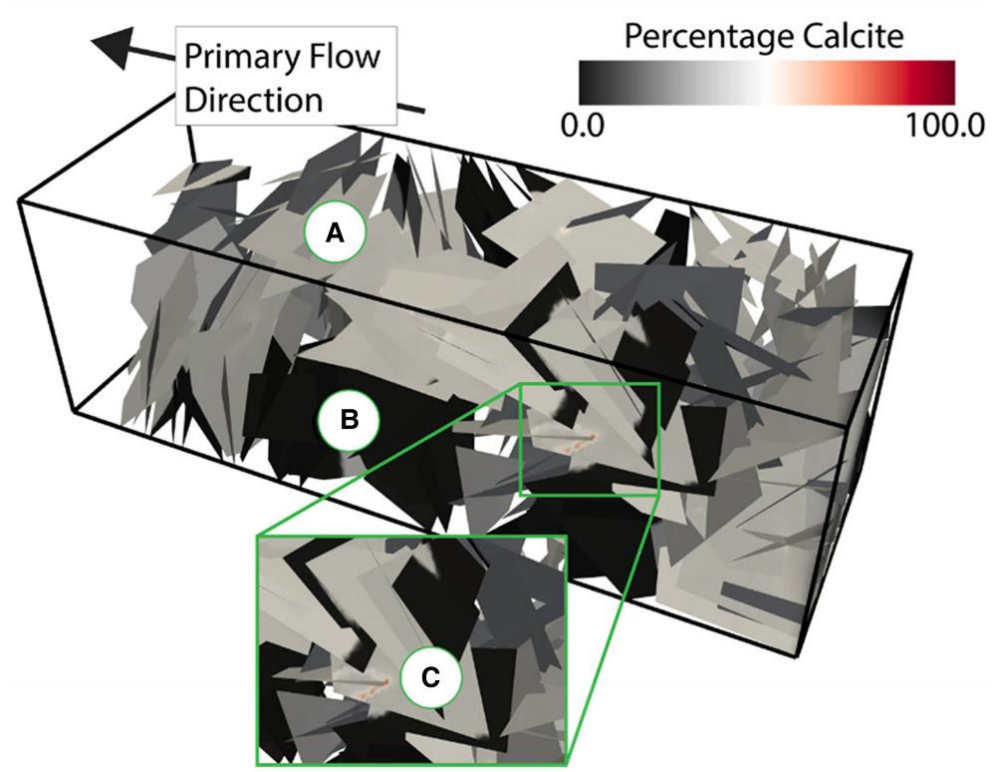
Calcite precipitation in a 3D fracture network. The spatially variable fluid flow field within the networks leads to heterogeneous calcite precipitation. A) Regions with a moderate amount of calcite (30–40% of the mesh cell is calcite). B) Regions with no calcite formation (0–5% of the mesh cell is calcite). C) A “hot spot” region with a lot of calcite precipitation (over 80% mesh cell is calcite).

Figure 9 shows the DFN with individual mesh cells colored by the percentage of calcite that has precipitated after 10 days. The DFN is initially filled uniformly with gypsum, and the initial flow field is heterogeneous both between fractures and within each fracture plane. This creates spatially variable intra-fracture and inter-fracture reaction conditions. Reactions within high-flow rate regions, which occur in the primary network channels, are kinetically limited. One example in this simulation is indicated by the (a) marker in the figure. Here, all of the gypsum has dissolved, and the cells are filled with around 30–40% calcite. In contrast, the reactions in low flow rate regions, in the secondary network, are transport-limited. One example in the simulations is indicated by the (b) marker in the figure. In this region, none of the gypsum has been dissolved and no calcite has precipitated. Finally, there are seemingly “hot spot” regions, indicated by the (c) marker in the figure, where a large amount of calcite has precipitated. These regions make up a very small portion of the DFN and tend to be in regions of flow confluence where the highest velocities occur.

These initial results demonstrate the delicate interplay of the complex fracture network structure, heterogeneous flow fields, and the resulting spatial variability of precipitation occurring at the network scale. Future work will focus on characterizing the specific geo-structural attributes that lead to this spatial variability in precipitation. In particular, understanding what leads to these “hot spot” formations is critical for carbon mineralization because they will be the primary region where clogging within the network would occur, which could lead to inefficiency or system collapse. Finally, extrapolating this model from the well-characterized gypsum system to fractured mafic/ultramafic rocks necessitates a thorough understanding and parameterization of mafic/ultramafic reactivity within flowing and stagnant fractures. Hence, the experimental and modeling approaches outlined in this study.

## Conclusions

The diverse experimental and modeling approaches employed in this study provide valuable insights into the complex process of carbon mineralization within fractured mafic and ultramafic rocks. By combining laboratory experiments and computational simulations, key factors influencing carbon mineralization rates and mechanisms can be more easily identified and leveraged to ensure sustainable, long-term carbon sequestration in these rock formations. Preliminary findings at the single fracture scale demonstrate the importance of coupled dissolution and precipitation processes in controlling reaction and flow dynamics. These include our observation of the differing impacts of surface roughness on iron oxide vs. MgCO_3_ precipitation, as well as the role of flow rate in controlling the relative abundance of different calcium carbonate polymorphs. While some of these phenomena can be captured by current models, we must advance these models to capture realistically complex geochemical environments to make defensible predictions of reactions that could influence the overall mineralization potential, such as surface passivation and reaction-driven cracking.

Current results from DFNs demonstrate that flow and reaction can be coupled in these complex models. However, they do not capture experimental observations: namely the presence of precipitation in dead-end fractures as observed in both our experiments and in the literature (34, 53, 54). Instead, the majority of precipitation was predicted to occur in regions with the highest flow velocities, while the dead ends contained neither gypsum dissolution nor calcite precipitation. It is possible that diffusion, dissolution, and precipitation rates utilized in current models are not sufficiently constrained by experimental data. However, this lack of precipitation more likely has to do with the time scales considered in the simulation rather than the model setup itself. Refinement of these variables is one future direction of our targeted experimental studies.

The observed rates of carbon mineralization in recent pilot tests, coupled with the abundance of suitable geological formations worldwide, suggest that carbon storage in mafic and ultramafic rock provides a viable pathway for mitigating anthropogenic carbon emissions and combating climate change. However, limited studies have focused on the mineralization processes within fractured rock. This comprehensive analysis provides a critical framework for considering carbon mineralization in fractured mafic and ultramafic rocks, offering valuable insights for both scientific understanding and practical applications in large-scale, long-term carbon sequestration operations.

## Data Availability

The main data supporting the findings of this study are available within the paper and cited references. PFLOTRAN (https://pflotran.org/) and dfnWorks (https://dfnworks.lanl.gov/) are open source and freely distributed simulation tools.
